# Membrane–Fabric Composite Filter Media for Continuous Cake Filtration without Gas Throughput Using Paste Dot Coating with Adhesive

**DOI:** 10.3390/membranes13090801

**Published:** 2023-09-18

**Authors:** Nikolai Benz, Fabian Krull, Kai Nikolaus, Sergiy Antonyuk

**Affiliations:** Institute of Particle Process Engineering, University of Kaiserslautern-Landau, 67663 Kaiserslautern, Germany

**Keywords:** cake filtration, cake deliquoring, membrane, composite, adhesive

## Abstract

In the field of liquid filtration, the realization of gas throughput-free cake filtration has been investigated for a long time. Cake filtration without gas throughput would lead to energy savings in general and would reduce the mechanically achievable residual moisture in filter cakes in particular. The reason why gas throughput-free filtration could not be realized with fabrics so far is that the achievable pore sizes are not small enough, and that the associated capillary pressure is too low for gas throughput-free filtration. Microporous membranes can prevent gas flow through open pores and cracks in the filter cake at a standard differential pressure for cake filtration of 0.8 bar due to their smaller pore size. Since large-scale implementation with membranes was not yet successful due to their inadequate mechanical strength, this work focuses on the development and testing of a novel composite material. It combines the advantages of gas throughput-free filtration using membranes with the mechanical stability of fabrics. For the production of the composites, a paste dot coating with adhesive, which is a common method in the textile industry, was used. Based on filtration experiments, delamination and tensile tests, as well as µCT analysis, it is shown that this method is suitable for the production of composite filter materials for gas throughput-free cake filtration.

## 1. Introduction

**Figure 1:**
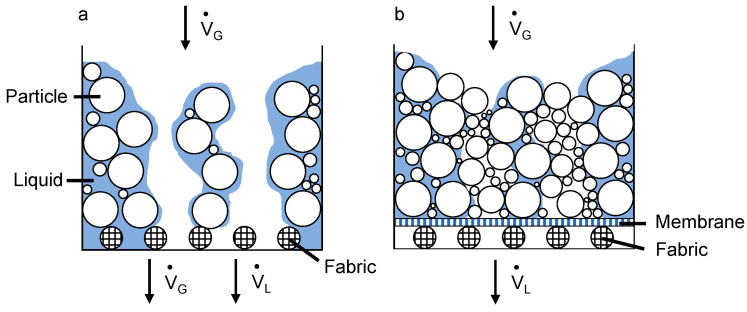
Filter cake deliquoring with (**a**) and without (**b**) gas throughput.

Cake filtration is a process to separate particles from liquid suspensions. The filtration can be performed with filters such as rotary drum filters and is driven by a vacuum inside the filter drum. The basic principle of the separation mechanism is that the filter drum covered with a filter medium rotates in a stirred suspension, the particles are deposited on the surface in the form of a filter cake and the filtrate is continuously discharged from the filter drum. After particle separation, the filter cake is washed, if necessary, dewatered and then ejected [1,2]. Filter cakes resulting from the filtration of concentrated suspensions in continuously operating filters are often dried thermally afterwards to obtain the dry product. This is the most expensive process step, because the remaining liquid has to be evaporated by means of heat supply. Filter cake deliquoring, such as gas differential pressure dewatering, can reduce the moisture in the filter cake and thus the energy required for thermal drying [3,4]. That is why the wish for gas throughput-free deliquoring on a large industrial scale has existed for decades. While the properties required for the filter medium and the operating parameters for the filter apparatus are sufficiently well known, the transition from a laboratory scale to larger industrially relevant scales has not been achieved, even at the present time [5,6,7]. In gas differential pressure deliquoring, the liquid present in the pore system of the filter cake is removed after overcoming the inlet capillary pressure, but for fine-grained cohesive particle systems, emptied pores and cracks in the filter cake occur and lead to an undesired gas throughput [8,9,10]. Figure 1 schematically shows the difference between filter cake deliquoring with gas throughput using fabric as a filter medium (Figure 1a) and filter cake deliquoring without gas throughput using a membrane–fabric composite (Figure 1b). Here, ${\dot{V}}_{G}$ describes the gas flow, and ${\dot{V}}_{L}$ describes the liquid flow.

The gas throughput has economic and technical disadvantages, as it must be compensated by the vacuum pump. Hence, gas throughput-free filtration would reduce not only the residual moisture in the filter cake, but also the necessary energy for thermal drying and pump losses due to vacuum breakdown. It would even be possible to operate the filtration completely without a vacuum pump and only by a hydrostatically induced pressure difference [6]. It has been shown in the past that the consolidation of the filter cake can lead to reduced cracking and also less residual moisture in the filter cake [11,12]. Consolidation results in a higher packing density of the particles, which makes it more difficult for cracks to form in the filter cake and thus also reduces the pore space between the particles in which water can accumulate. However, such technical adaptations of the filter apparatus would not be necessary when realizing gas throughput-free filtration by means of filter elements. Usually for the filtration of numerous particle systems, a wide variety of filter media with different mesh and pore sizes are used, which can be subdivided in general into woven fabrics, nonwovens and membranes [13,14]. While membranes are used primarily for separating the finest particle sizes, woven fabrics are designed to have long service lives and high filtrate throughputs. Due to their high mechanical strength, they are common filter materials for drum filters. It has already been shown that besides the separation of the finest particle fractions, microporous membranes based on polymers or ceramics can ensure gas throughput-free cake filtration [15,16,17,18]. These microporous properties are the basis of why the composite filter medium ensures filtration without gas throughput (Figure 1b). The necessary capillary pressure, respectively the nominal pore size of the membrane required for gas throughput-free filtration can be estimated (Equation (1)).

(1) $$p_{k}=(4\cdot \gamma \cdot \cos\delta )\cdot d^{-1}$$

Here, *γ* is the surface tension, *δ* is the wetting angle, d is the pore diameter, and $p_{k}$ is the capillary pressure [19]. If the membrane capillary pressure is higher than usual differential pressures for filter cake dewatering (0.8 bar), the liquid can be removed from the filter cake but not from the membrane pores. Therefore, the result is gas throughput-free cake filtration [5,7,18]. However, the disadvantage is that microporous membranes do not have sufficient mechanical strength to be used on large industrial drum filters, contrary to fabrics. On the other hand, it is not possible to produce fabrics or nonwoven materials with the pore sizes required for gas throughput-free cake filtration as these are limited due to the production process and especially the fiber thicknesses used. In this work, a filter medium made of a woven filter cloth and a microporous membrane is presented that can avoid gas throughput and has the potential to quickly go beyond the laboratory scale through the use of point-bonded lamination processes [20]. On the basis of filtration experiments, delamination tests, tensile tests and µCT analysis, it has been shown that this method is generally suitable for the production of composite filter materials for gas throughput-free cake filtration. With its microporous properties, the membrane is responsible for preventing gas throughput and will also predominantly define the filtration properties in general. The supporting fabric underneath stabilizes the membrane and thus should finally enable its implementation on large-scale apparatuses.

## 2. Materials and Methods

### 2.1. Filter Media

For the membrane–fabric composite filter media, two different fabrics (SEFAR TETEX MULTI 05-4-660 K PHARMA and SEFAR TETEX MULTI 05-130-200 W) and a microporous membrane (3M MicroPES 12F) were used. The composite based on the first aforementioned fabric was primarily used to gain experience and to be able to test the handling of the experiments beforehand. The second was used for the final composite prototype. The fabrics are multifilaments made of polypropylene (PP) and differ in their thickness, area weight and permeability. The membrane is made of polyether sulfone (PES). With a bubble point of 1.05 bar in water, the membrane is suitable for ensuring gas throughput-free filtration and deliquoring at a standard differential pressure of 0.8 bar. Due to its small thickness, the membrane provides simultaneous high throughputs for its nominal pore size of approx. 1 µm [21]. The properties of the fabrics and the membrane are summarized in Table 1 and Table 2. The values listed there were taken from the data sheets of the respective manufacturers.

**Figure 2:**
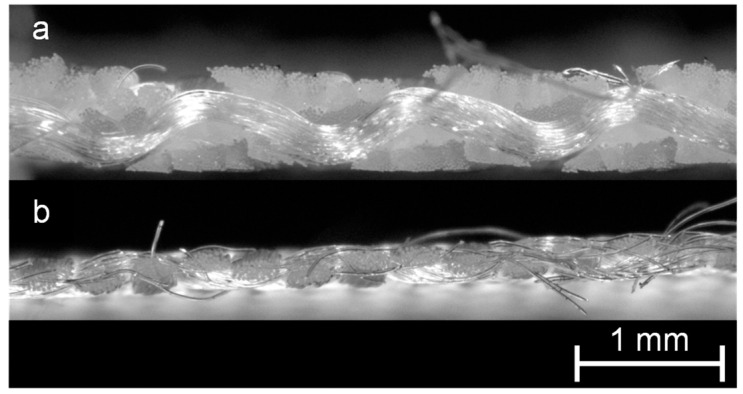
Microstructure of the fabric 05-4-660 K PHARMA (**a**) and 05-130-200 W (**b**).

**Figure 3:**
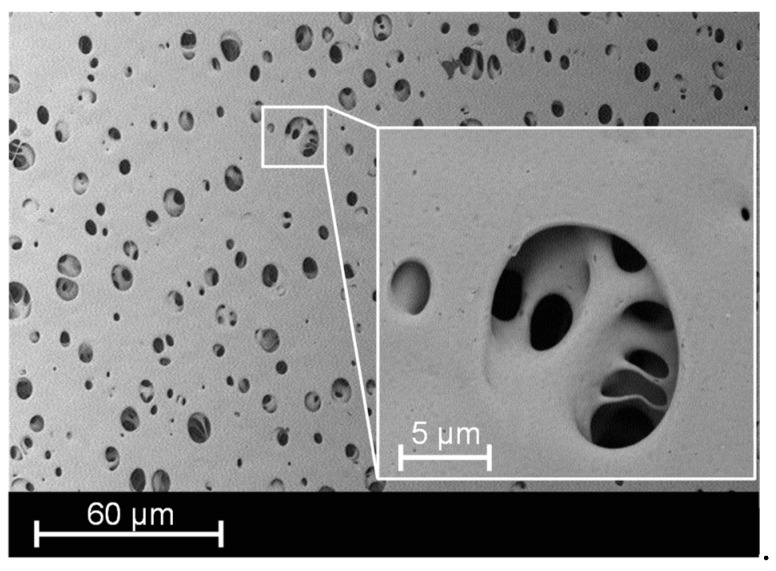
SEM image of the surface of the membrane MicroPES 12F.

In Figure 2, microscope images of the cross-sectional area of the fabric 05-4-660 K PHARMA (Figure 2a) and of the fabric 05-130-200 W (Figure 2b) are shown. In Figure 3, an SEM image of the surface of the membrane MicroPES 12F is shown.

### 2.2. Paste Dot Coating with Adhesive

**Figure 4:**
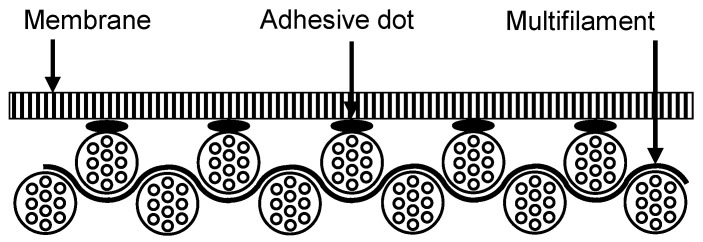
Schematic structure of the membrane–fabric composite produced by paste dot coating with adhesive.

In general, paste dot coating with adhesive or point-bonding is a manufacturing process that is well known in the textile industry and is already being applicable on a large industrial scale [22,23,24,25,26]. This process is used to join two or more layers of textile materials together by applying adhesive dots. The adhesive dots are applied on one side of the textile by means of a rotating screen, while the textile layer to be joined is added and pressed on afterward [27]. The amount of adhesive can be adjusted by the hole size of the screen, the squeeze pressure, the rotation speed of the screen and the viscosity of the adhesive [23]. Paste dot coating is particular used for manufacturing breathable textiles and sportswear because the advantage of point-bonding is that the quantity of adhesive application simultaneously influences the ratio of joint strength to free area [28]. This correlation is also investigated in the production of the membrane–fabric composites for gas throughput-free cake filtration. In addition to the adhesion properties between membrane and fabric, the flow resistance in particular is decisive when used for filtration applications. Since the flow resistance should be directly related to the free area and the amount of adhesive blocking the membrane and fabric pores, the best possible balance must be found between these parameters. A schematic of the membrane–fabric composites produced is shown in Figure 4. For the bonding, a polyurethane hotmelt adhesive was used.

The actual production of the composite materials was carried out by the company Trans-Textil GmbH, Freilassing, Germany, which uses this process for the production of textiles and clothes. The sample material was made from DIN A4-sized pieces of the fabrics 05-4-660 K PHARMA and 05-130-200 W and the membrane MicroPES 12F.

### 2.3. Determination of the Filter Medium Resistance

**Figure 5:**
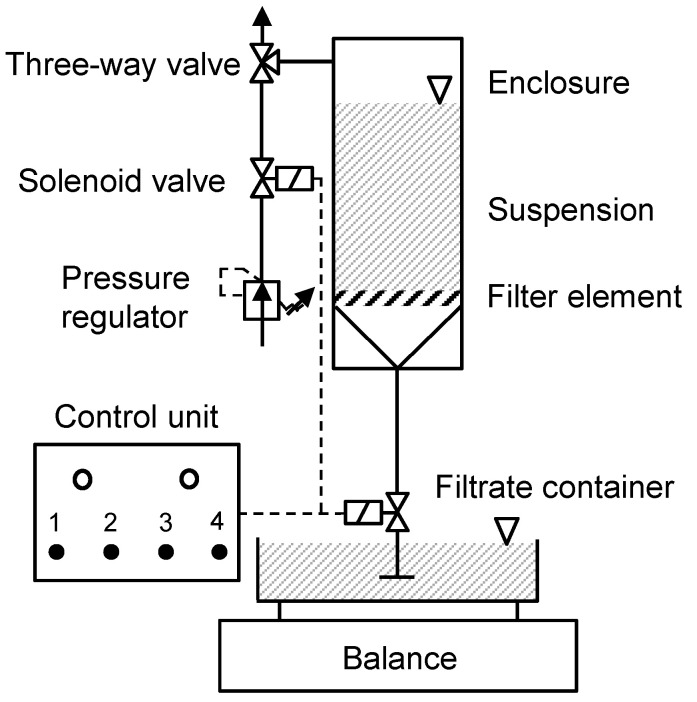
Improved pressurized filter cell according to VDI 2762.

The challenge in manufacturing the composites is that the adhesive dots should not significantly increase the filter medium resistance $R_{M}$ by blocking individual pores to ensure maximum possible filtrate flow. Compared to woven filter elements, membranes already have a lower maximum possible flow rate due to their smaller pore sizes, which should not be further minimized. Otherwise, the lack of throughput alone would make large-scale implementation unprofitable. In order to determine the influence of the surface coverage, as well as the associated pore blockage caused by the adhesive dots on the filter medium resistance, flow-through tests were carried out with the pressurized filter cell according to VDI 2762 with 250 mL demineralized (DI) water at a differential pressure of 0.8 bar [29]. To ensure an accurate determination, the improved experimental setup with respect to the measurement resolution developed at the institute was used [30]. The schematic structure of the improved pressurized filter cell is shown in Figure 5.

Compared to the standard measuring setup, solenoid valves are used here to enable precise pressure application and thus a precise start of the actual filtration. The balance (WIPOTEC EC3000) also offers an increased resolution of 0.02 g at time intervals of 10 ms. The effects of the resolution improvement on the filtration results, as well as a detailed description of the measurement setup can be taken from the published paper [30]. During the measurement the filtrate mass is recorded, converted into volume V over time t, and plotted in a t/V-V diagram. From the slope and the y-axis intercept of the resulting straight line, the filter medium resistance $R_{M}$ is determined using Equation (2) from the VDI 2762 [29]. Here, η is the dynamic viscosity of water, k is the concentration parameter, ∆p is the set differential pressure, A is the filter area in the pressurized filter cell, and $\alpha_{H}$ is the filter cake resistance, which can only be determined for particle filtrations.

(2) $$\frac{t}{V}=\frac{k\cdot \eta \cdot \alpha_{H}}{2\cdot A^{2}\cdot ∆p}\;V+\frac{\eta \cdot R_{M}}{A\cdot ∆p}$$

### 2.4. Micro-Computed Tomography Imaging/Operating Principle and Image Creation

Micro-computed tomography (µCT) images were made to localize the exact position of the adhesive dots in the composite material. In general, computed tomography is used to examine objects to obtain information about their composition and internal structure [31]. Computed tomography works with X-rays that irradiate the object. The X-rays interact with the materials of the sample and a detector measures the transmission of the X-ray beams [32]. In contrast to radiography, the sample in µCT scans is positioned on a rotary table, which rotates the sample 360° in small steps during the measurement. This creates a large number of radiographic images in small angular steps. These projection images are three-dimensionally reconstructed so that a volumetric image of the sample is calculated [33,34]. The evaluation of the raw data of the measurement is carried out using sectional images of the reconstructed volume, which show pixels with gray values. The brightness of the gray values correlates with the attenuation of the X-rays measured at the detector and caused by interaction with the sample material. This correlation can be described via the Beer–Lambert law. It describes the attenuation of the radiation intensity $I\left(x\right)$ from the initial intensity $I_{0}$ by an exponential decrease with the thickness of the sample x and the attenuation coefficient *μ* [32,35].

$$I\left(x\right)=I_{0}e^{-\mu x}$$

The µCT sectional images consist of gray values that represent the respective locally prevailing attenuation coefficient *μ*. The dependence of the attenuation coefficient and the direct proportionality to the material density is used in the subsequent evaluation of the sectional images for segmentation. The µCT images from the composite filter media were taken using a TomoScope^®^ L 300 (Werth Messtechnik GmbH, Giessen, Germany) with a transmission X-ray tube and a 4K detector with 100 µm pixels. The µCT measurements were made at X-ray tube parameters of 90 kV and 40 W. The integration time was 134 ms. Three measurements were repeated per rotation step to average the signal. The scanned volume was 1971 × 1971 × 1282 voxels with a voxel size of 12.32 µm. The specimens had dimensions of 50 × 70 mm, which is the maximum sample size for that kind of material that can still be measured well in the µCT used without the loss of resolution. The largest measuring area possible is necessary to guarantee a statistically reliable statement about the distribution of the adhesive dots. To verify the set voxel size of 12.32 µm for a sample size of 50 × 70 mm, µCT images of a small sample were made once with a voxel size of 2.45 µm and once with a voxel size of 12.32 µm. Both resolutions and the resulting sectional images were then compared with regard to the total solid area fractions calculated using MATLAB. An SEM image of a single adhesive dot was also taken and compared to the projection area of the same adhesive dot from the µCT images to verify the set voxel size again.

### 2.5. Delamination and Tensile Tests

**Figure 6:**
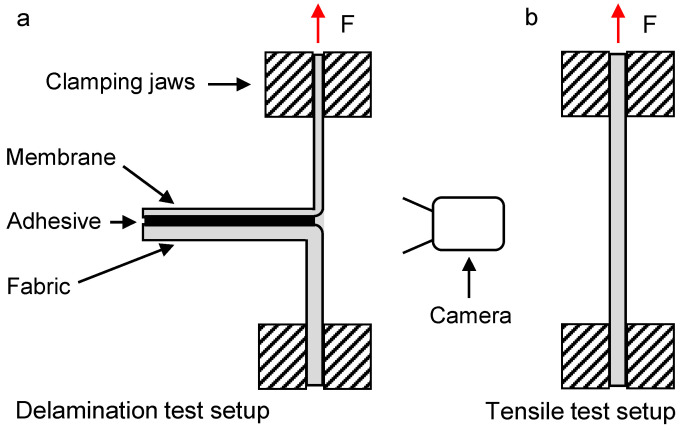
Test setup for the delamination (**a**) and tensile (**b**) tests, where the applied force F is marked with a red arrow.

In order to determine the bond strength between the membrane and the fabric, delamination tests were carried out using a Texture Analyzer TA.XTplus from Stable Micro Systems, Godalming, UK. The experimental setup for the delamination tests with the composites is shown in Figure 6a, where F is the applied force.

During the testing, the membrane side was attached to the upper clamping jaw, and the fabric side was attached to the lower clamping jaw, which is statically connected to the bottom of the testing machine. Then, the mounted specimen was stretched under a small load. Using a camera, the detachment of the two layers at the breakage surface was recorded in parallel with the force and displacement measurement. The obtained breakage force of the composite was also compared with the breakage force of the membrane, which was determined via a uniaxial tensile test (Figure 6b). The reason for this is to determine whether delamination forces in the range of the maximum tensile force of the membrane can be achieved. This is the limiting factor for testing and real application, as the bond strength can only be determined if it is below the tensile strengths of the two materials bonded together. Although a joint strength above the maximum tensile strength of the membrane would be theoretically possible, it would not play a decisive role for real application cases, since the membrane itself would fail beforehand and gas throughput would not be reliably prevented by a damaged membrane layer. All tests were performed at an experimental speed of 0.1 mm/s. The Texture Analyzer TA.XTplus has a displacement resolution of 1 µm and a force resolution of 1 mN. The specimens were cut out of the bonded composites at different locations in order to obtain representative samples for the entire lamination process. The specimens had dimensions of 10 × 50 mm. Delamination tests were performed for both versions of the point bonded membrane–fabric composites. The sample wide for the tensile tests was also 10 mm with a free clamping length of 20 mm.

## 3. Results

### 3.1. Filter Medium Resistances

**Figure 7:**
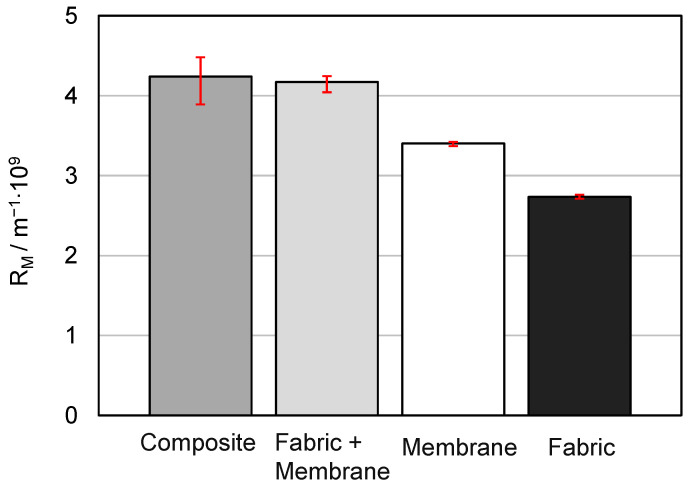
Comparison of the filter medium resistances.

The measured filter medium resistances are shown in Figure 7. Here, the abbreviation “Fabric” stands for the fabric 05-130-200 W; “Fabric + Membrane”, for the fabric 05-130-200 W with the non-bonded membrane (MicroPES 12F) on top; “Membrane”, for the membrane MicroPES 12F; and “Composite”, for the spot-bonded composite filter media made out of the fabric 05-130-200 W and the membrane MicroPES 12F.

As can be seen from the measurement results, the filter medium resistance of the membrane that is point-bonded to the support fabric is only slightly higher compared to the filter medium resistance of the membrane lying on the support fabric. Since identical production batches were used in the selection of the test materials and the flow-through tests were carried out under identical laboratory conditions, the differences between these two measuring values can be attributed exclusively to the point-bonding. It can therefore be stated that the point-bonding has a negligibly influence on the filter medium resistance overall. The explanation for this could be that during the production of the composite material, the adhesive dots were applied in areas that are not relevant to the flow. Thus, the adhesive dots were most likely placed predominantly on the top of the fibers. Even if the fabric is a multifilament in which the fabric fibers themselves consist of individual fibers, it can be assumed that the multifilament fiber itself is not flowed through or, similarly, the flow rate through the fiber is negligible compared to the flow rate around the fiber. So as long as the adhesive dots do not block the flow areas between the individual multifilament fibers, a significant increase in the filter medium resistance is not expected. Since the membrane used is a microporous membrane in which flow is not limited to the main flow direction, the adhesive dots can also be flowed around inside the membrane. This could be another explanation why the flow resistance is not significantly increased.

### 3.2. Micro-Computed Tomography Analysis

#### 3.2.1. Verification of the Measurement Resolution

**Figure 8:**
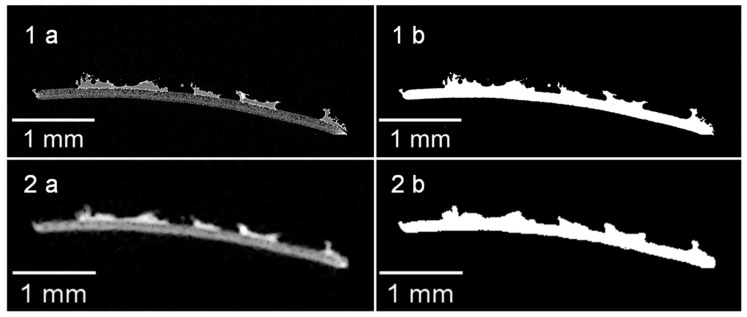
Sectional images of the membrane with adhesive dots on the surface taken at different resolutions ((**1a**)—voxel size: 2.45 µm; (**2a**)—voxel size: 12.32 µm) with the marked solid area fractions by means of threshold values (**1b**,**2b**).

To investigate the microstructure of the produced membrane–fabric composite, µCT measurements were applied, whereby the influence of the used voxel size on the accuracy of the microstructure data was first evaluated. To achieve this, a sample of the membrane with the adhesive dots on the surface, which originated from a previously performed delamination test according to Section 2.5, was measured with two different voxel resolutions (2.45 µm and 12.32 µm). In Figure 8 the cross-sectional images of the µCT measurements are shown with the corresponding solid sample area marked in white. Here, the Figure 8(1a) shows the cross-sectional image of the sample taken with a voxel size of 2.45 µm, Figure 8(1b) shows the associated marked solid area fraction, Figure 8(2a) shows the same sample measured with a voxel size of 12.32 µm, and Figure 8(2b) again shows the associated marked solid area fraction.

**Figure 9:**
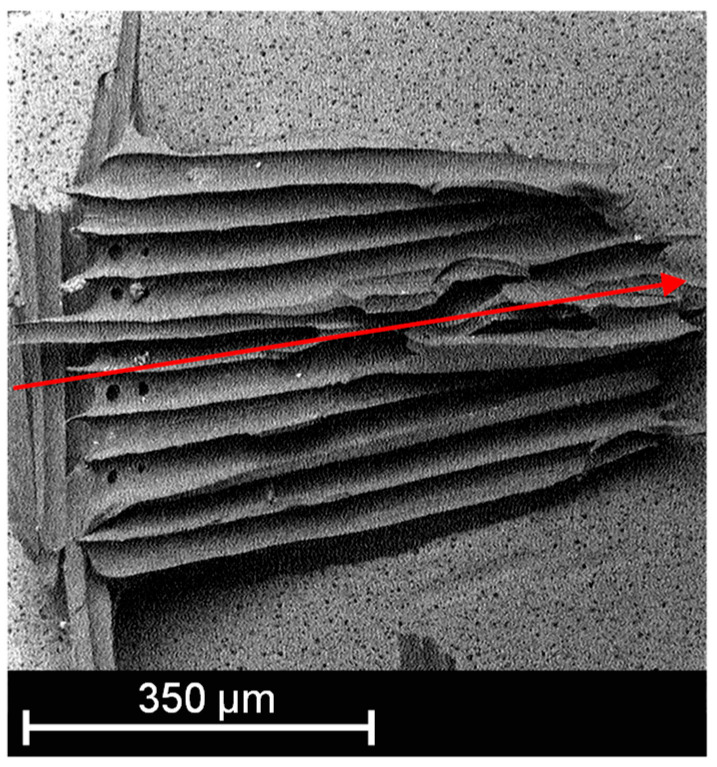
Single adhesive dot on the membrane surface recorded with SEM, (red arrow: length of the adhesive dot).

It became clear according to Figure 8 that a smaller voxel size can achieve a higher level of detail. On the basis of the white marked solid area fractions in Figure 8, the total area was calculated for comparability purposes. For a set voxel size of 2.45 µm (Figure 8(1b)), the area is 0.5870 mm^2^ and 0.6031 mm^2^ for a set voxel size of 12.32 µm (Figure 8(2b)). This results in a deviation of 2.67%. In addition, the length of a single adhesive dot was compared by means of an SEM image with the µCT images taken beforehand. The SEM image of an adhesive dot is shown in Figure 9.

**Figure 10:**
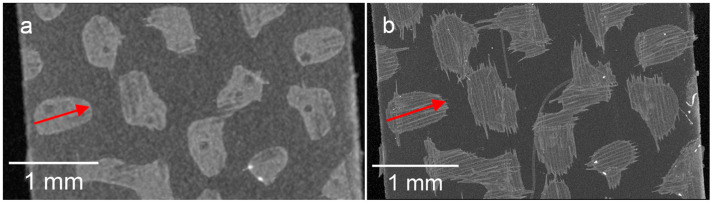
Surface of the membrane with the adhesive dots measured with a voxel size of 12.32 µm (**a**) and with a voxel size of 2.45 µm (**b**), (red arrow: length of the adhesive dot).

The surface of the membrane with the adhesive dots on top reconstructed from the µCT images is shown in Figure 10.

The ImageJ freeware software (version 1.53t) was used to determine the length of the adhesive dots in each figure (red arrows). On the basis of the measurement results, the length of the adhesive dot recorded with SEM is 0.707 mm based on the reconstruction of the µCT 0.661 mm for a voxel size of 12.32 µm and 0.709 mm for a voxel size of 2.45 µm. It becomes clear that with a smaller voxel size, the length can be determined almost similarly compared to SEM. In contrast, the length deviation of the same adhesive dot determined between the µCT measurements is 6.77%. However, in order to obtain a reliable statement about the adhesive dot distribution, the largest sample possible should be measured in the µCT, which is only possible for a specimen size of 50 × 70 mm with a set voxel size of 12.32 µm. Therefore, the error caused by the reduced resolution can be accepted in favor of a higher statistical significance. In general, it must be considered that the deviation between measurements can also be explained by the evaluation method, in which the boundary between the adhesive and membrane is determined. A generally valid definition of gray values for the adhesive and membrane is not possible due to the different measurement principles and interferences in optical measurement methods. However, for the prevailing adhesive dot sizes in the range of 500–1500 µm, the resulting error arising from the lower resolution is considered negligible since the statistical resilience of the adhesive dot distribution is of primary importance.

#### 3.2.2. Determination of the Adhesive Dot Distribution

**Figure 11:**
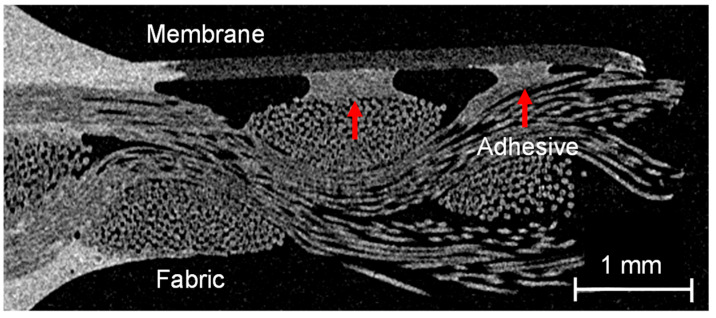
Sectional image of the membrane–fabric composite with the fabric 05-4-660 K PHARMA and the membrane MicroPES 12F (red arrows: adhesive dots).

To confirm the assumption that the adhesive dots are predominantly located on the upstanding fabric fibers, the µCT images of the produced material were evaluated. A sectional image of the membrane–fabric composite with the fabric 05-4-660 K PHARMA and the membrane MicroPES 12F is shown in Figure 11.

**Figure 12:**
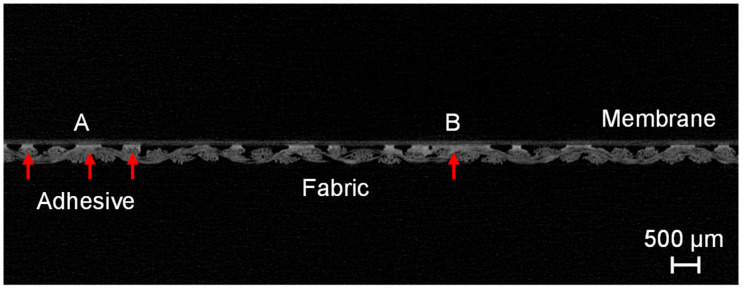
Sectional image of the membrane–fabric composite with the fabric 05-130-200 W and the membrane MicroPES 12F (red arrows: adhesive dots).

The individual components of the composite material can be well differentiated in the µCT scan on the basis of the gray value differences. The adhesive dots are highlighted with red arrows in Figure 11. On the basis of the µCT images, it becomes clear that during the manufacturing process with the coarser fabric (05-4-660 K PHARMA), the connection by means of adhesive dots with the membrane was not always ideal due to the strongly deviating surface structure of the two materials. As long as the adhesive dots are directly on top of the multifilament fiber there is no loss of adhesion. However, due to the coarser weave, the adhesive dots are sometimes placed more laterally on the multifilament fiber. This increases the distance to the underside of the membrane, which must be bridged by the adhesive dots to ensure sufficient adhesion. Bridging the distance, and thereby ensuring bond strength, can be achieved either by increasing the adhesive quantity per dots, by increasing the quantity of dots per area, or by a combination of both. The µCT images also show that the adhesive dots spread out along the fiber directions when the second layer is rolled up. This results in the merging of several small adhesive dots into larger ones on the fiber tops. When the adhesive is applied and the second layer is pressed on by means of a roller, it is ensured that the contact pressure acts predominantly on the fabric fibers lying on top. Thus, when the appropriate amount of adhesive per area is selected the probability of adhesive dots between the fibers blocking the flow is minimized. Blocking with adhesive only occurs if too much adhesive or too-high contact pressures prevail and the formation of a closed adhesive layer is possible. This can also be the case if higher adhesive quantities or higher contact pressures only prevail locally due to manufacturing tolerances. A sectional image of the membrane–fabric composite with the fabric 05-130-200 W and the membrane MicroPES 12F is shown in Figure 12.

Here, A indicates the area where the adhesive dots are ideally applied to the fiber on the upper side, and B shows a local spread of the adhesive across three fiber bundles. When using the fabric 05-130-200 W, it has been shown that a more uniform distribution of adhesive dots is generally possible due to the previously mentioned effects.

### 3.3. Image Analysis and Determination of the Surface Coverage by the Adhesive Dots

**Figure 13:**
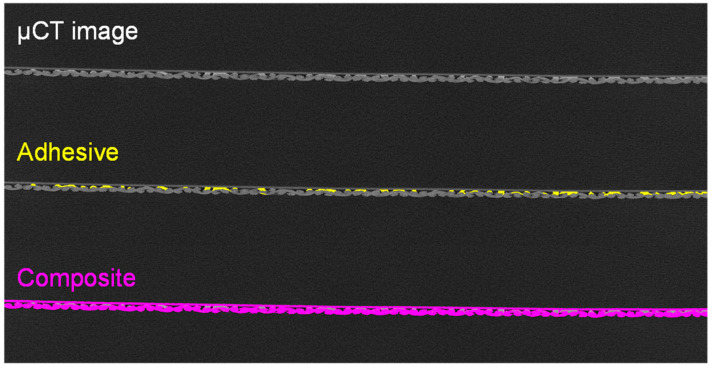
Defining threshold values for the evaluation of the µCT measurements.

The emerging gray value differences between fabric, membrane, and adhesive were used to calculate the percentage distribution of the individual components in relation to the entire sample. The calculation was performed by defining the upper and lower threshold values for the respective gray values of the composite components (Figure 13).

**Figure 14:**
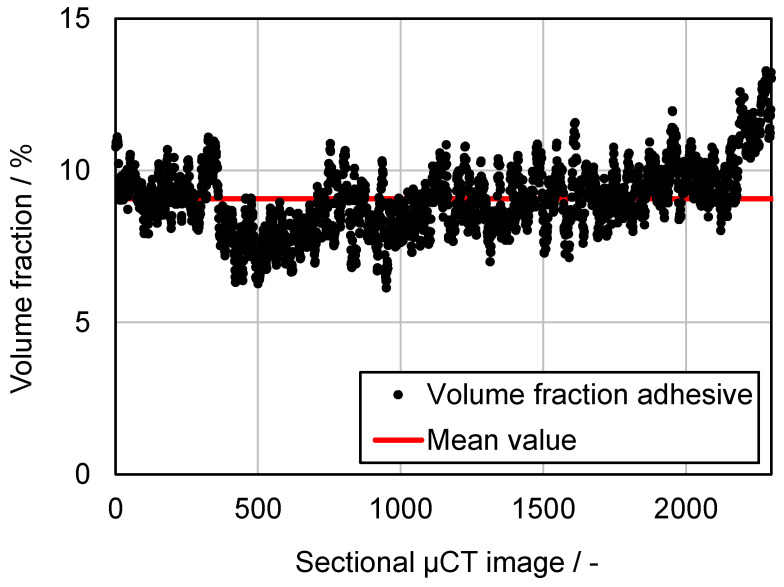
Volume fraction of adhesive per sectional image over the entire specimen.

Figure 13 shows an original sectional image of the µCT measurement (top), the same image with the adhesive points highlighted after the definition of the threshold values (middle), and the whole solid volume of the sample highlighted (bottom). The obtained volume fraction of the adhesive in the composite evaluated based on the µCT measurement is shown in Figure 14.

**Figure 15:**
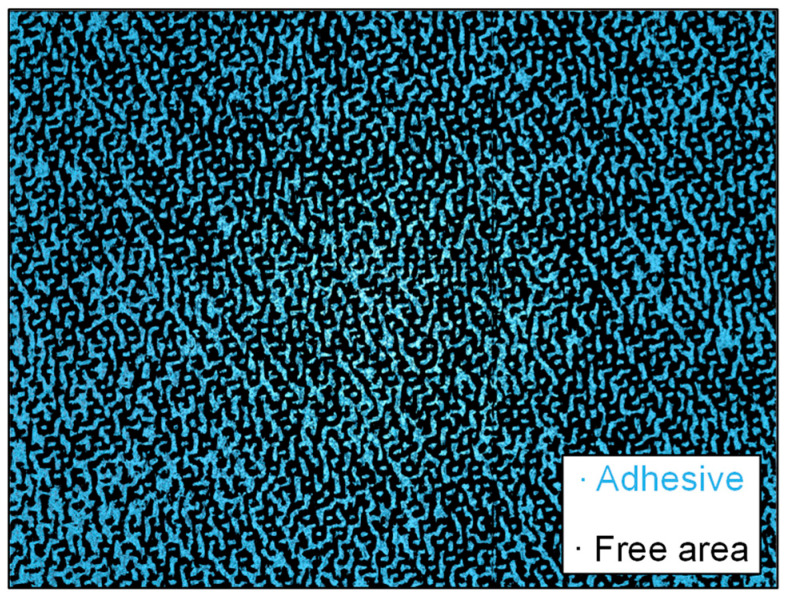
Surface distribution between adhesive and free area (view: upstream side).

The calculation of the adhesive proportion was carried out for each individual image. Since the cross-sectional images of the specimen show the distance of the set voxel size from each other, the area fraction can also be seen as the volume fraction of the adhesive. The adhesive volume is therefore the adhesive area multiplied by the depth (one voxel). From the evaluated data, it becomes clear that a uniform adhesive application has been achieved during the production of the composite material because the volume fraction only deviates by a few percent across all cross-sectional images of the specimen. As shown in Figure 14, the applied adhesive has an average volume fraction of 8% of the total composite volume, as shown in Figure 13. However, the calculated area fractions in the cross-sections do not correspond with the covered area from the upstream side. In this case, only the spreading of the adhesive dots on the fibers is decisive, as it can block the flow directly. For this reason, the µCT images were re-evaluated with the projection in the direction of the membrane surface. This resulted in the surface distribution shown in Figure 15.

In Figure 15, all areas colored in blue correspond to the adhesive dots, and those in black represent the free area without adhesive application. The distribution shows that the adhesive dots are aligned on the respective fibers. The theoretical coverage of the upstream side by the adhesive dots is almost 50%, but as previously explained, even a high coverage of the incident flow surface does not significantly hinder the flow resistance as long as they are not predominantly located in areas relevant for the flow.

### 3.4. Delamination and Tensile Tests of the Membrane–Fabric Composites

**Figure 16:**
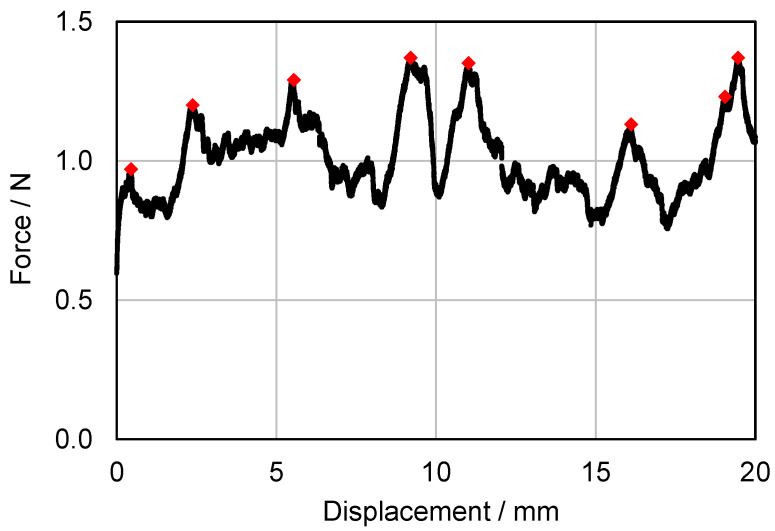
Typical force–displacement diagram measured in a delamination test.

Figure 16 shows a typical force–displacement diagram of the delamination tests described in Section 2.5.

**Figure 17:**
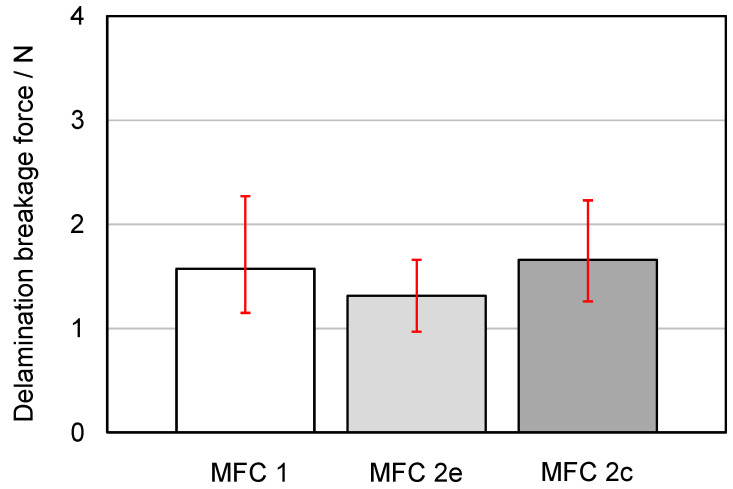
Delamination breakage force of the composites measured with the test setup according to Figure 6a.

For the evaluation of the individual measurements, the peaks over the measurement distance were considered and averaged over the number of measurements. The peaks (red dots in Figure 16) can be assumed to represent the failure of a complete row of adhesive dots lying next to each other. A series of small force peaks or an associated uniform application of force is more likely to indicate that individual sections of the adhesive dots failed one after the other or that the joint was not optimal at the given point. The results of all delamination tests are shown in Figure 17.

**Figure 18:**
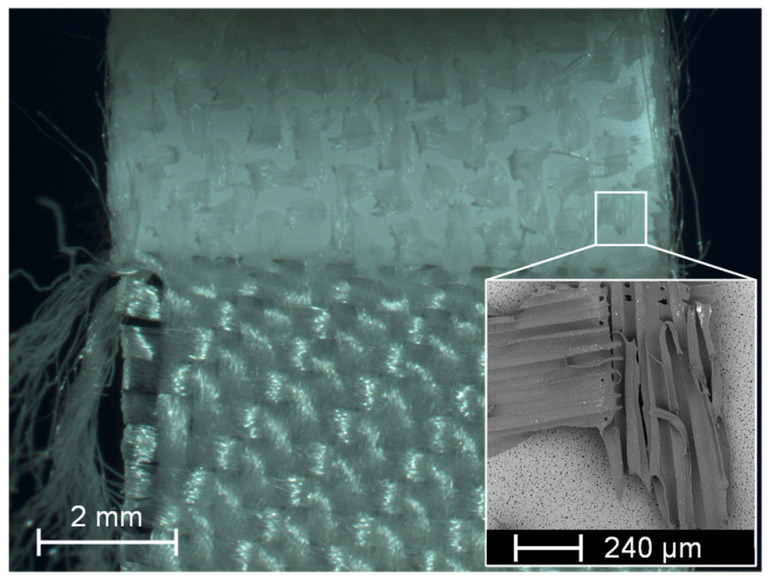
Image of the breakage surface with a SEM image of the adhesive dot.

The abbreviation “MFC 1” stands for the composite with the fabric 05-4-660 K PHARMA and the membrane MicroPES 12F; the abbreviation “MFC 2”, for the composite with the fabric 05-130-200 W and the membrane MicroPES 12F; “e”, for samples taken at the edge; and “c”, for samples taken at the center. An image of the breakage surface with a SEM close-up of a single adhesive dot on the membrane side is shown in Figure 18.

**Figure 19:**
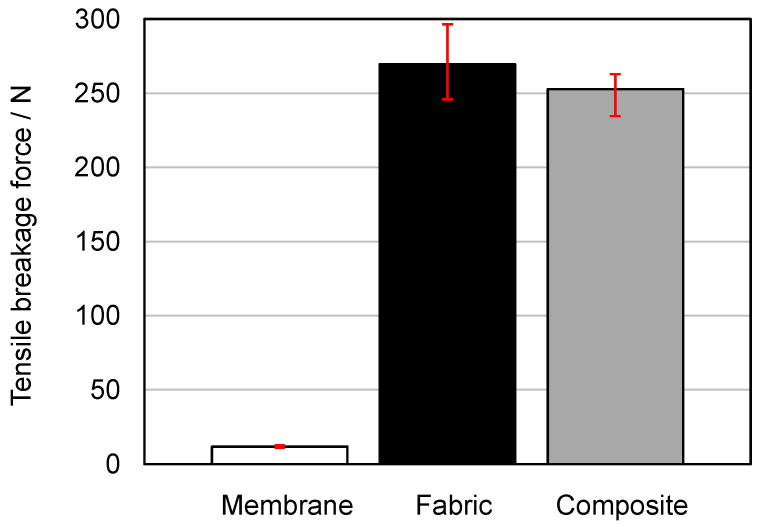
Tensile breakage forces of the membrane MicroPES 12F, the fabric 05-130-200 W, and the composite measured with the test setup according to Figure 6b.

As can be seen in Figure 17, only slight differences exist between the delamination breakage forces required to delaminate the composite components at the edge and in the center of the material exists. This confirms a homogeneous manufacturing process with an associated uniform distribution of the adhesive dots, as could already be determined on the basis of the µCT images. Also, the type of fabric, i.e., whether it has a coarse or finely woven surface structure, has been shown to play a subordinate role with regard to the adhesion properties as long as the adhesive dots were properly applied. The parallel recording of the breakage surface with the camera as well as the SEM recording of a single adhesive dot provided extended information on the adhesion properties. As can be seen in Figure 18, the detachment of the adhesive dots took place exclusively on the fabric side. The adhesion properties of the used polyurethane-based hotmelt adhesive are therefore better for the PES membrane than for the PP fabric. This also became evident from the SEM image, which clearly showed that the individual fiber bundles of the multifilament have been released from the adhesive while the adhesive remains on the membrane. In Figure 18, it is also clearly visible that the elongated orientation of the adhesive dots on the underside of the membrane can be attributed to the direction of the fabric fiber at the corresponding opposite contact point. Hence, the finding that the adhesive dots are located predominantly on the upper side of the multifilament fibers could be confirmed again. The results of the tensile tests according to the test setup in Figure 6b are shown in Figure 19.

The abbreviation “Membrane” stands for the membrane MicroPES 12F; “Fabric”, for the fabric 05-130-200 W; and “Composite”, for the composite with the fabric 05-130-200 W and the membrane MicroPES 12F. As can be seen in Figure 19, the tensile breakage force of the composite is predominantly influenced by the tensile breakage force of the fabric used. The resistance of the membrane to mechanical tensile load is several times lower. This was also the basic assumption and motivation for the production of the composite materials, which has now been confirmed once again by the measurements. The fact that the composite has, on average, slightly lower tensile breakage forces than the fabric can be attributed to either manufacturing tolerances in weaving or minor deviations in cutting the specimens to size. When comparing the mean values of the delamination breakage force of the composite “MFC2” in Figure 17 at the edge (1.31 N) and in the center (1.66 N) with the mean tensile breakage force of the membrane (12.08 N), it becomes clear that further optimization potential for the bond strength between the membrane and fabric is possible since maximum delamination breakage forces can only be achieved in the range of the tensile breakage forces of the weakest component of the composite, i.e., the membrane.

## 4. Conclusions

For several decades, investigations have been focused on the realization of gas throughput-free cake filtration, whereby the filter media in particular remains a critical part for successful large-scale industrial implementation. In this study, a novel membrane–fabric composite filter medium was developed and tested with the goal of enabling large-scale application due to the manufacturing process used. Paste dot coating with adhesive, a known method in the textile industry, was applied to bond a PES membrane to a PP fabric. The selected PES membrane ensures continuous cake filtration without gas throughput at a standard differential pressure of 0.8 bar and, at the same time, a maximum possible flow rate for this pore size.

Based on flow through measurements, it was shown that a paste dot coating with adhesive can be used for filtration applications without significantly hindering the flow. This is crucial for industrial filtration since membranes generally have a lower flow-through rate than fabrics due to their smaller pore sizes. Therefore, the maximum possible flow rate of the membrane should not be significantly reduced by the application of an adhesive. In the worst-case scenario, flow rates that are too low can make the entire process uneconomical. Why the filter medium resistance is not greatly affected by the adhesive dots could be explained using the µCT images. After evaluation of the individual µCT sectional images, it becomes clear that the adhesive dots was able to be predominantly applied to the fabric fibers standing upwards due to the contact pressure of the application roller. At these locations, the adhesive dots do not impede the flow since the flow predominantly passes around the fabric fibers or the flow rate through a multifilament fiber is negligibly small. Based on the delamination tests carried out, it was also shown that a homogeneous composite production could be achieved, as the delamination breakage force did not show any major changes over different samples. The aim behind the production of the composite material, which was to reinforce the membrane with a fabric, was also demonstrated by the tensile tests performed. It became clear that the overall strength of the composite was predominantly influenced by the fabric used. In summary, on the basis of the various experiments performed, it can be stated that the membrane–fabric composites produced have the potential to quickly leave the laboratory scale and finally the ensure large-scale industrial implementation of gas throughput-free cake filtration.

For future membrane–fabric composites, bonding identical material pairings would be interesting as it can be assumed that bonding a PES fabric to a PES membrane would result in higher adhesion properties and higher delamination breakage forces. Due to the lack of availability of PES fabric, this assumption has yet to be investigated in detail because commercially available fabrics for filtration are predominantly made out of PP. Identical material combinations would be also beneficial with regard to the chemical resistance of the membrane–fabric composite since each individual material has different resistances that must be considered in combination with each other.

The feasibility of a point-bonded membrane–fabric composite and the associated advantages and challenges in manufacturing, which were discussed in this study, offers overall the possibility of initiating new product developments for manufacturers of filtration materials. In terms of further expected cost increases for energy and necessary resource conservation in general, efforts to apply an industrially applicable filter medium for gas throughput-free cake filtration should still be regarded as worthwhile.

## Figures and Tables

**Table 1:** Properties of the fabrics.

Product Name	Material	Thickness/µm	Area Weight/g∙m^−2^	Permeability/ (L/m^2^)/s at 200 Pa
05-4-660 K PHARMA	PP	1060	660	4
05-130-200 W	PP	450	200	167

**Table 2:** Properties of the membrane.

Product Name	Material	Thickness/µm	Bubble Point (in Water)/bar	Transmembrane Flow/ mL/(min cm² bar)
MicroPES 12F	PES	110 ± 10	1.05 ± 0.25	260

## Data Availability

Not applicable.
